# Proteomic analysis of tumor necrosis factor-α resistant human breast cancer cells reveals a MEK5/Erk5-mediated epithelial-mesenchymal transition phenotype

**DOI:** 10.1186/bcr2210

**Published:** 2008-12-16

**Authors:** Changhua Zhou, Ashley M Nitschke, Wei Xiong, Qiang Zhang, Yan Tang, Michael Bloch, Steven Elliott, Yun Zhu, Lindsey Bazzone, David Yu, Christopher B Weldon, Rachel Schiff, John A McLachlan, Barbara S Beckman, Thomas E Wiese, Kenneth P Nephew, Bin Shan, Matthew E Burow, Guangdi Wang

**Affiliations:** 1 Department of Chemistry, Xavier University of Louisiana, New Orleans, LA 70125, USA; 2 Chengdu Blood Center, Chengdu, Sichuan 610041, PR China; 3 Department of Medicine, Tulane University School of Medicine, Tulane Avenue, New Orleans, LA 70112, USA; 4 Center for Bioenvironmental Research at Tulane and Xavier Universities, Tulane Avenue, New Orleans, LA 70112, USA; 5 Tulane Cancer Center, Tulane Avenue, New Orleans, LA 70112, USA; 6 Department of Pharmacology, Tulane University School of Medicine, Tulane Avenue, New Orleans, LA 70112, USA; 7 Department of Surgery, Tulane University School of Medicine, Tulane Avenue, New Orleans, LA 70112, USA; 8 Department of Surgery, Children's Hospital Boston, Longwood Avenue, Boston, MA 02115, USA; 9 The Dan L Duncan Cancer Center, Baylor College of Medicine, Baylor Plaza, Houston, TX 77030, USA; 10 College of Pharmacy, Xavier University of Louisiana, New Orleans, LA 70125, USA; 11 Medical Sciences, Indiana University School of Medicine, East 3rd Street, Bloomington, IN 47405-4401, USA

## Abstract

**Introduction:**

Despite intensive study of the mechanisms of chemotherapeutic drug resistance in human breast cancer, few reports have systematically investigated the mechanisms that underlie resistance to the chemotherapy-sensitizing agent tumor necrosis factor (TNF)-α. Additionally, the relationship between TNF-α resistance mediated by MEK5/Erk5 signaling and epithelial-mesenchymal transition (EMT), a process associated with promotion of invasion, metastasis, and recurrence in breast cancer, has not previously been investigated.

**Methods:**

To compare differences in the proteome of the TNF-α resistant MCF-7 breast cancer cell line MCF-7-MEK5 (in which TNF-α resistance is mediated by MEK5/Erk5 signaling) and its parental TNF-a sensitive MCF-7 cell line MCF-7-VEC, two-dimensional gel electrophoresis and high performance capillary liquid chromatography coupled with tandem mass spectrometry approaches were used. Differential protein expression was verified at the transcriptional level using RT-PCR assays. An EMT phenotype was confirmed using immunofluorescence staining and gene expression analyses. A short hairpin RNA strategy targeting Erk5 was utilized to investigate the requirement for the MEK/Erk5 pathway in EMT.

**Results:**

Proteomic analyses and PCR assays were used to identify and confirm differential expression of proteins. In MCF-7-MEK5 versus MCF-7-VEC cells, vimentin (VIM), glutathione-S-transferase P (GSTP1), and creatine kinase B-type (CKB) were upregulated, and keratin 8 (KRT8), keratin 19 (KRT19) and glutathione-S-transferase Mu 3 (GSTM3) were downregulated. Morphology and immunofluorescence staining for E-cadherin and vimentin revealed an EMT phenotype in the MCF-7-MEK5 cells. Furthermore, EMT regulatory genes SNAI2 (slug), ZEB1 (δ-EF1), and N-cadherin (CDH2) were upregulated, whereas E-cadherin (CDH1) was downregulated in MCF-7-MEK5 cells versus MCF-7-VEC cells. RNA interference targeting of Erk5 reversed MEK5-mediated EMT gene expression.

**Conclusions:**

This study demonstrates that MEK5 over-expression promotes a TNF-α resistance phenotype associated with distinct proteomic changes (upregulation of VIM/*vim*, GSTP1/*gstp1*, and CKB/*ckb*; and downregulation of KRT8/*krt8*, KRT19/*krt19*, and GSTM3/*gstm3*). We further demonstrate that MEK5-mediated progression to an EMT phenotype is dependent upon intact Erk5 and associated with upregulation of SNAI2 and ZEB1 expression.

## Introduction

Drug resistance represents a major obstacle to successful therapy of breast cancer, a leading cause of death among women in Western countries [1]. It is well known that several ATP-binding cassette transporters, such as MDR (multidrug resistance), MRP (multidrug resistance associated protein), and BCRP (breast cancer resistance protein), are related to the development of drug resistance in breast cancers [2-4]. However, many other proteins – including glutathione-S-transferase [5], β_2_-microglobulin [6], heat shock protein (HSP)27 [7,8], 14-3-3σ [9,10], and vimentin [11] – have also been implicated in breast cancer drug resistance. These findings were based upon studies using various chemoresistant breast cancer cell lines such as adriamycin, verapamil, tamoxifen, vinblastine, and paclitaxel resistant MCF-7 cells. Although some aspects of the mechanisms of drug resistance have been characterized, the highly variable response to chemotherapy in the treatment of breast cancers remains poorly understood. Elucidating these drug resistance mechanisms is essential for improving tumor responses to clinical chemotherapies.

A growing area of interest that may reveal one such mechanism is the association of drug resistance with epithelial-mesenchymal transition (EMT) in cancer. EMT is the process by which adherent epithelial cells convert to motile mesenchymal cells and is essential in embryonic development. However, it appears that aberrant activation of EMT occurs in cancer progression [12], and is involved in highly aggressive, poorly differentiated breast cancers with increased potential for metastasis and recurrence [13]. EMT has been linked to resistance to various drugs in cancer, including tamoxifen resistance in breast carcinoma cells [14], paclitaxel resistance in epithelial ovarian carcinoma cells [15], oxaliplatin resistance in colorectal cancer cells [16], gemcitibine resistance in pancreatic tumor cells [17], cetuximab resistance in hepatoma cells [18], and erlotinib resistance in non-small-cell lung carcinomas [19]. The activities of several genes are known to contribute to EMT, including decreased expression of E-cadherin, and increased expression of snail, slug, and δ-EF1 (ZEB1) [20]. Increased expression of vimentin [21] and N-cadherin [22] are also seen in EMT. Evaluation of these markers in a drug-resistant cell line may shed light on the relationship between EMT and drug resistance.

TNF-α is a multifunctional cytokine that elicits a variety of biologic responses, such as inflammation and apoptosis [23]. Additionally, TNF-α has been shown to induce EMT [24,25]. Although TNF-α is not currently an anticancer agent for treatment of human cancers (because of side effects such as normal cell toxicity), low doses of TNF-α can markedly sensitize cancer cells to chemotherapy-induced apoptosis [26,27]. We previously demonstrated that MCF-7 cell line variants exhibit differences in sensitivity to TNF-α and apoptosis induced by taxol and doxorubicin [28-30]. Specifically, we demonstrated that apoptosis sensitive MCF-7-N cells (MCF-7 N variant) exhibited distinct differences in cell survival and apoptotic signaling when compared with inherently resistant MCF-7-M cells (MCF-7 M variant) [28]. We further demonstrated that apoptosis sensitive cells (MCF-7-N) could be driven to a resistant phenotype through prolonged exposure to increasing concentrations of TNF-α, leading to a stable, apoptosis-resistant phenotype (MCF-7-TNR) that was in part dependent upon mitogen-activated protein kinase (MAPK) and nuclear factor-κB signaling [29]. Gene expression profiling revealed that MAPK kinase (MEK)5 was over-expressed in the TNF-α resistant MCF-7-M cells versus the TNF-α sensitive MCF-7-N cells [31]. A similar upregulation of MEK5 in resistant cells was independently described in MCF-7-F cells, which developed resistance to the pure anti-estrogen fulvestrant through prolonged growth in fulvestrant-containing media [32]. These studies demonstrate a potential role for the MEK5 pathway in the regulation of progression to drug resistance in breast cancer.

The MEK5/extracellular signal-regulated kinase (Erk)5 tandem is a component of MAPK cascades that mediate signals from various extracellular stimuli to the nucleus and regulate most cellular processes [33], including gene expression, proliferation, apoptosis, and motility [34,35]. MAPK signaling may also play a role in EMT [12,36]. Although MEK5/Erk5 signaling has not been extensively investigated, several studies suggest a role in cancer progression. For example, MEK5/Erk5 signaling has been demonstrated in prostate and breast cancer proliferation and tumorigenesis [37,38]. Furthermore, inhibition of MEK5/Erk5 signaling in the MDA-MB-231 cell line, an aggressive breast cancer cell line with an EMT phenotype, induces apoptosis [39].

Based on these findings, which strongly indicate that MEK5/Erk5 signaling may mediate cancer progression to an aggressive phenotype, we further explored the involvement of MEK5/Erk5 signaling in resistance to apoptosis as well as EMT. To test the role played by MEK5/Erk5 activation in progression of breast carcinoma cells to a resistant phenotype, MCF-7 cells (N variant) were used to stably express a constitutive active MEK5 construct. These MCF-7-MEK5 cells exhibit resistance to TNF-α as compared with stable vector cells (MCF-7-VEC). Proteomic analysis based on two-dimensional electrophoresis (2-DE) and various mass spectrometric techniques has been employed in several studies of drug resistance of breast cancers [8-10,40]. In this study we used a proteomics approach to define mechanisms of the MEK5 signaling cascade in the regulation of drug resistance. The differentially expressed proteins identified in proteomic analyses were confirmed at the gene expression level using reverse transcription RT-PCR assays. Results using immunofluorescence staining and gene expression analysis were consistent with an EMT phenotype in the MCF-7-MEK5 cells. These findings identify a potential role for the MEK5 pathway in coordinately promoting both an EMT phenotype and TNF-α resistance in breast cancer.

## Materials and methods

### Stable transfection of constitutively active MEK5

The DNA expression-construct pCMV-HA-CA-MEK5 [41] was used to derive MEK5 over-expressing variants of MCF-7 (N variant) cell line and selected with G418 (Gibco, Paisley, UK). A total of 5 × 10^6 ^MCF-7 cells were plated in 10 mm dishes in mammary epithelial growth medium and 10% Dulbecco's modified Eagle's medium, and incubated in 5% carbon dioxide at a temperature of 37°C. The following day, cells were co-transfected with pCMV-HA-CA-MEK5/pCMVdsRED-Neo at a 4:1 ratio using FuGENE (Roche, Nutley, NJ), in accordance with the manufacturer's instructions. Stable transfectants were selected by culturing cells in the presence of 200 μg/ml G418 (drug levels maintained during culture of cell stocks but removed for individual experiments). Medium was removed and replaced with fresh medium every 3 days until visible clones appeared. Individual clones were isolated using a sterile cloning ring coated with petroleum jelly and removed with phosphate-buffered saline (PBS) containing 1 mmol/l EDTA. These clones, called MCF-7-MEK5, were transferred to 24-well plates and allowed to grow as separate clones in mammary epithelial growth medium and 10% Dulbecco's modified Eagle's medium. MCF-7-VEC was established simultaneously as a stable, vector control cell clone. For RNA interference studies we used an Erk5 targeting pSUPER-EGFP PolymeraseIII driven RNA interference system (generously provided by Dr Frank E Jones, University of Colorado, Denver, CO, USA). Optimized hairpin RNA interference sequences for Erk5 were identified using OligoEngine software (Oligoengine, Seattle, WA, USA). The pSuper Erk5 EGFP clone was generated by ligating the annealed oligo with *Sal*I-*Bgl*II digested pSuper-EGFP vector and confirmed by sequencing.

### Western blotting assay for MEK5 and Erk5

MCF-7-MEK5 and MCF-7-VEC cells were characterized by immunoblotting with antibodies against MEK5 and Erk5. Western blots of crude whole cell extracts were performed using standard procedures. About 5 × 10^6 ^cells were harvested in sonicated buffer for 30 seconds and collected by centrifugation at 1,000 *g *for 20 minutes. Fifty micrograms of protein was resuspended in sample loading buffer, boiled for 5 minutes, and electrophoresed on polyacrylamide gel. The proteins were transferred electrophoretically to a nitrocellulose membrane, blocked with PBS-Tween (0.05%) in 5% low-fat dry milk solution at room temperature for 1 hour, incubated with specific antibodies (overnight at 4°C), and then washed in PBS-Tween solution three times. Then the immunoreactive proteins were visualized using an electrogenerated chemiluminescence system (Amersham, Arlington Heights, IL, USA) and recorded by fluorography on Hyperfilm (Amersham), in accordance with the manufacturer's instructions.

### Colony survival assay for TNF-α in the established MCF-7-MEK5 cells

Clonogenicity assays were performed as we have described previously [42,43]. For TNF-α colony survival assay, MCF-7-MEK5 and MCF-7-VEC cells were plated and treated with different concentrations of TNF-α (0 to 100 ng/ml) for 18 hours. Cells were then cultured in fresh media without TNF-α and observed daily for 1 to 2 weeks. Colonies were fixed, stained with crystal violet and counted. Data are represented as percentage clonogenic survival from untreated control cells (100%) ± standard error of the mean (n = 3).

### Immunofluorescence analysis of EMT markers and morphology

The expression levels of an epithelial cell marker (E-cadherin) and a mesenchymal cell marker (vimentin) were assessed by indirect immunofluorescence using specific antibodies (E-cadherin: CS-3195 [Cell Signaling Technology, Beverly, MA, USA]; vimentin: V6630 [Sigma, St. Louis, MO, USA]). The distribution of filamentous actin (F-actin) was visualized using Alexa 488 conjugated phalloidin (Invitrogen, Carlsbad, CA, USA) [44]. Briefly, MCF-7, MCF-7-MEK5, and MDA-MB-231 cells were cultured in eight-well chamber slides for 48 hours. The cells were fixed in 4% paraformaldhyde/PBS for 10 minutes followed by incubation with the primary antibodies and phalloidin at the desired dilution (E-cadherin: 1:50 dilution; vimentin: 1:50 dilution; phalloidin: 1:100 dilution). Alexa 594 and 488 conjugated secondary antibodies (1:1,500 dilution) were used to detect E-cadherin and vimentin, respectively. The nucleus was stained using DAPI containing VectorShield mounting medium (Vector Laboratories, Burlingame, CA, USA). The digital images were captured using Nikon Eclipse 80*i *along with the accompanying program IPLab, version 3.6.5 (Nikon Inc., Melville, NY, USA).

### 2-DE and image analysis

A total of 1 × 10^7 ^cells for each cell line (MCF-7-MEK5 and MCF-7-VEC) were collected and homogenized in lysis buffer (7 mol/l urea, 2 mol/l thiourea, 4% 3-[(3-Cholamidopropyl)dimethylammonio]-1-propanesulfonate (CHAPS), and 50 mmol/l dithiothreitol (DTT)) containing protease inhibitor cocktail (Sigma), and sonicated three times for 7 seconds each. The method of Bradford (BioRad, Hercules, CA, USA) was used to detect the protein concentrations, and the samples were stored at -70°C for further analyses.

First dimensional electrophoresis was performed using a Protean iso-electronic focus cell unit (BioRad). Precast 11 cm immobilized pH gradient (IPG) strips with pH ranges 5 to 8 were obtained from BioRad. Lysates were thawed and mixed (3:1) with rehydration solution (7 mol/l urea, 2 mol/l thiourea, 4% CHAPS, 50 mmol/l DDT, 5% Triton ×100, 5% ampholytes, and 0.01% of bromophenol blue). Two hundred microliters (approximately 180 μg) of protein sample mixture was loaded to each of the IPG strips. The strips were rehydrated with the sample mixture and overlaid with a layer of mineral oil overnight (16 hours). Iso-electric focusing was carried out in three steps under gradient mode: 250 V for 20 minutes, linear ramp; 8,000 V for 2.5 hours, linear ramp; and 8,000 V, rapid ramp to reach 20,000 V-hours.

The second dimensional electrophoresis was carried out in a BioRad Criterion electrophoresis cell system. IPG strips were equilibrated in two steps: 20 minutes in 60 mmol/l DTT and 20 minutes in 200 mmol/l of iodoacetamide; both were dissolved in equilibration buffer (6 mol/l urea, 1% SDS, 30% glycerol, and 40 mmol/l Tris-base). IPG strips were sealed on the top of SDS gels (Criterion 8% to 16% Tris-HCl Gel obtained from BioRad) with 0.5% agarose. SDS-PAGE was performed at a constant voltage of 200 V for 55 minutes. Gels were washed with 200 ml de-ionized water three times for 5 minutes each. Bio-safe staining solution (50 ml; BioRad) was added to each gel, and the gels were then placed on a shaker for 2 hours. The staining solution was discarded and the stained gels were rinsed with de-ionized water (3×, 30 minutes per wash). Stained gels were scanned with a Gel Doc-XR image system (BioRad) and analyzed with the PDQuest software (version 8.01) (Bio-Rad, Hercules, CA, USA). The proteins of interest were marked for excision. The spots were excised from the gels by a Quest Spot cutter (BioRad), and digested with trypsin (Promega, Madison, WI, USA) in 25 mmol/l ammonium bicarbonate overnight at 37°C to release tryptic peptides using a Progest digestion system (Genomic Solutions, Ann Arbor, MI, USA). Recovered peptides were dried in a Labconco NewCentriVap concentrator (Labconco, Kansas City, MO, USA).

### Liquid chromatography tandem mass spectrometry analyses and data processing

The dried protein digests from the 96-well plates were analyzed using an LC-Nanospray-MS^n ^system (Thermo Scientific, Waltham, MA, USA), equipped with a Finnigan Micromass AS automatic sampling system. A BioBasic C18 PicoFrit Column (75 μm × 10.2 cm, tip 15 μm; New Objective, Woburn, MA, USA) was used for separation. The mobile phases consisted of (A) 0.1% formic acid in water and (B) 0.1% formic acid in acetonitrile, at a linear gradient from 0% B to 35% B within 35 minutes, along with a gradient from 35% B to 98% B within 8 minutes, kept at 98% B for 2 minutes and then back to 0% B within 2 minutes. The total acquisition time was 60 minutes for each run. The tandem mass spectrometry spectra were analyzed against the ipi.human.v3.27 database using SEQUEST software (Thermo Scientific, Waltham, MA, USA), and the results were tabulated for each identified protein.

### Reverse transcription polymerase chain reaction

Total RNAs from MCF-7-MEK5 and MCF-7-VEC cells were extracted using a PureLink total RNA purification system (Invitrogen) or RNeasy^® ^mini kit (Qiagen Sciences, Germantown, MD, USA). The reverse transcription was carried out with a SuperScript first-strand synthesis system (Invitrogen) using Oligo(dT)_12–18 _primers or with iscript™ cDNA synthesis kit (Biorad Laboratories, Hercules, CA USA). The primer pairs used to amplify the genes *vim *(vimentin), *krt8 *(keratin 8), *krt19*, *hspa4 *(HSPA4), *gstp1 *(glutathione-S-transferase P), *gstm3 *(glutathione-S-transferase Mu 3) and *ckb *(creatine kinase B-type), and the genes involved in EMT including *cdh1 *(E-cadherin), *ctnnb1 *(β-catenin), *snail*, *slug *and *δ-ef1 *(δ-EF1) were designed using the online tool of Oligo Perfect Designer (Invitrogen), and the endogenous *actb *(β-actin) was employed as an internal standard. The primer pairs are provided in the additional materials [see Additional data file 1]. Primer specificity was confirmed by BLAST (Basic Local Alignment Search Tool) analysis. Standard PCR was performed using a Platinum blue PCR supermix kit (Invitrogen). Briefly, denaturation was carried out at 94°C for 2 minutes, and 30 cycles (94°C for 30 seconds, 58°C for 30 seconds, and 72°C for 30 seconds), followed by a 5 minute period for elongation at 72°C. The PCR products were isolated by 1.5% agarose gel electrophoresis, and the bands were visualized by SYBR green staining. For real-time PCR analyses, an iCycler iQ5 real time PCR detection system (BioRad), and a SYBR GreenER qPCR supermix kit (Invitrogen) were used as follows: 50°C for 2 minutes, 95°C for 8 minutes and 30 seconds, and 50 cycles (15 seconds at 95°C, 1 minute at 60°C). The data were analyzed with a normalized gene expression method (ΔΔ Ct) [45] using the iQ5 Optical System Software (BioRad), and the gene *actb *was used as a reference for normalization. All experiments were repeated three times independently.

## Results

### MEK5/Erk5 activation promotes resistance to TNF-α

**Figure 1:**
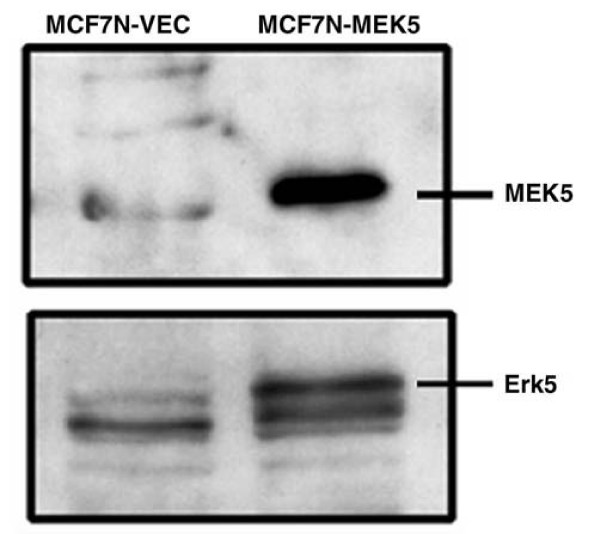
**Generation of stable MCF-7-MEK5 cells**. Stable clones of MCF-7 cells expressing either constitutively active MEK5 (MCF-7-MEK5) or vector (MCF-7-VEC) were examined by Western blot analysis for expression of MEK5 (upper panel) and Erk5 (bottom panel). Erk, extracellular signal-regulated kinase; MEK, mitogen-activated protein kinase kinase.

**Figure 2:**
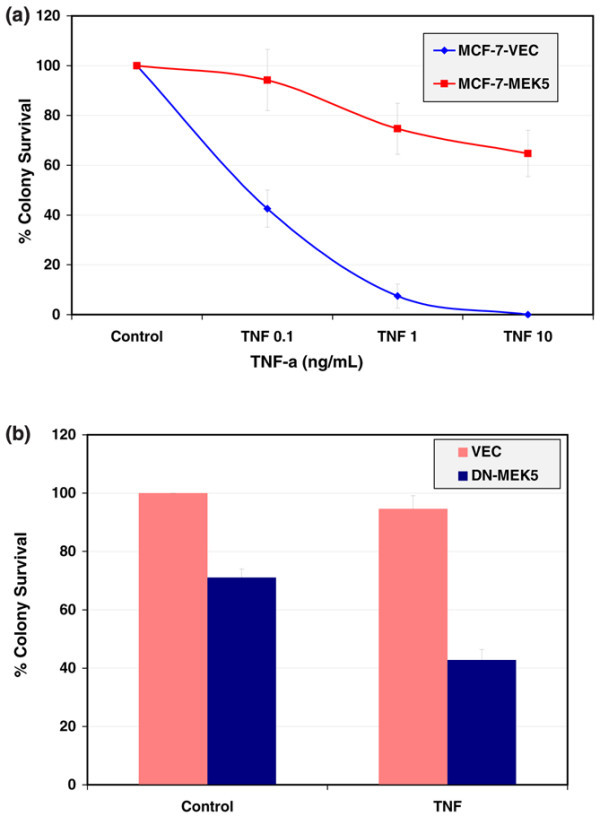
**CA-MEK5-mediated resistance to TNF-α induced loss of clonogenic survival**. **(a) **MCF-7-MEK5 and MCF-7-VEC cells were plated for clonogenic survival assay and treated with different concentrations of TNF-α (0 to 100 ng/ml) for 18 hours. Following this, cells were cultured in a fresh media without TNF-α and observed daily for 1 to 2 weeks. **(b) **MCF-7-MEK5 cells were plated for clonogenic survival assay and transfected with vector or DN-MEK5 (100 ng/well) for 24 hours. The following day cells were treated with vehicle or TNF-α (1 ng/ml) for 18 hours. Following this, cells were cultured in a fresh media without TNF-α and observed daily for 1 to 2 weeks. Colonies were fixed, stained with crystal violet and counted. Data are presented as percentage clonogenic survival from untreated control cells (100%) ± standard error of the mean (n = 3). MEK, mitogen-activated protein kinase kinase; TNF, tumor necrosis factor.

Stable clones of MCF-7-VEC parental and its CA-MEK5 over-expressing cells, MCF-7-MEK5, were characterized by immunoblotting with antibodies against MEK5 and Erk5 (Figure 1). We assayed *in vitro *colony formation of MCF-7-VEC and MEK5 cells in response to TNF-α treatment. Cells were fixed and stained after 10 (MEK5) or 15 days (VEC) and stained with crystal violet. Then the number of colonies was counted and normalized to the control (= 100%). As shown in Figure 2a, MCF-7-VEC was sensitive to TNF-α in a dose dependent manner, being unable to form colonies at a final concentration of 10 ng TNF-α/ml. In contrast, the capacity of MEK5 cells to form colonies was only reduced to 64.7 ± 9.3% as compared with controls. To confirm the requirement for MEK5 in this resistance, MCF-7-MEK5 cells were transiently transfected with empty vector (VEC) or a dominant negative MEK5 mutant construct (DN-MEK5; Figure 2b). In vector transfected groups, treatment with TNF resulted in 94.6 ± 4.4% colony formation as compared with control cells. Transfection with DN-MEK5 reduced colony formation to 42.8 ± 3.6% with TNF treatment as compared with control. These results demonstrate that a DN-MEK5 partially suppresses basal clonogenicity as well as enhancing sensitivity to TNF-α.

### Differences in protein expressions characterized by proteomic analysis

**Figure 3:**
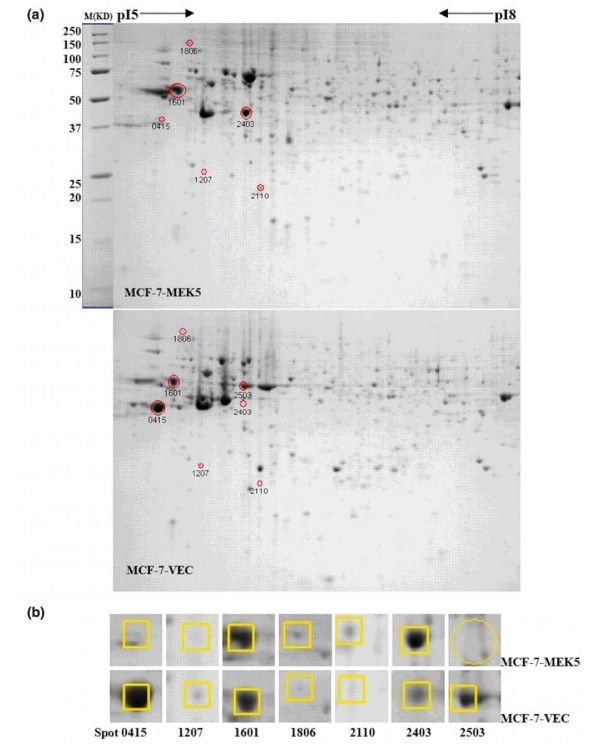
**2-DE images of MCF-7-MEK5 and MCF-7-VEC cells**. **(a) **2-DE image of the total proteins extracted from MCF-7-MEK5 and MCF-7-VEC cells. **(b) **A total of seven differentially expressed protein spots were identified, and the changes in protein expressions are presented in Table 1. 2-DE, two-dimensional electrophoresis; M, molecular weight; MEK, mitogen-activated protein kinase kinase; pI, isoelectric pH.

Proteome analysis was performed to compare the differences in protein expression by examining whole-cell protein extractions obtained from MCF-7-MEK5 and MCF-7-VEC cells. Following 2-DE and Coomassie blue staining, the gels were analyzed using the PDQuest image analysis software in triplicate experiments and subjected to capillary liquid chromatography-tandem mass spectrometry analysis. A total of 56 protein spots were identified that differed significantly (more than twofold difference) in the two cell lines, of which seven proteins were recognized as most relevant to the present study (Figure 3 and Table 1). These protein spots yielded rich peptide fragments and were found to have similar theoretical and experimental molecular weights and isoelectric pH values. The seven proteins selected for further investigation from the comparative 2-DE analysis were KRT19, GSTM3, VIM, HSPA4, GSTP1, CKB, and KRT8. In MCF-7-MEK5 cells, VIM, HSPA4, GSTP1 and CKB were upregulated, whereas KRT8, KRT19 and GSTM3 were downregulated as compared with MCF-7-VEC cells. Indeed, expression of KRT8 in MCF-7-MEK5 cells was below the detection limit under the current proteomics conditions. Other differentially expressed proteins observed in proteomic analysis are described separately the additional materials [see Additional data file 2].

**Table 1:** Identification of the seven protein spots with liquid chromatography-tandem mass spectrometry

Spot number	Protein symbol	Theoretical MW (kDa)	Experimental MW (kDa)	Theoretical pI	Experimental pI	Ratio (MCF-7-MEK5 versus MCF-7-VEC)
0415	Keratin 19 (KRT19)	44.07	46	4.9	5.2	0.01
1207	Glutathione-S-transferase Mu 3 (GSTM3)	26.54	26	5.25	5.7	0.37
1601	Vimentin (VIM)	53.62	55	4.91	5.2	2.14
1806	Heat shock 70 kDa protein 4 (HSPA4)	94.24	100	5.03	5.3	1.81
2110	Glutathione-S-transferase P (GSTP1)	23.34	23	5.32	5.8	5.41
2403	Creatine kinase B-type (CKB)	42.62	45	5.25	5.7	57.67
2503	Keratin 8 (KRT8)	53.67	50	5.38	5.8	0

The spot number corresponds to that reported in Figure 3. MW, molecular weight; pI, isoelectric pH.

### Confirmation with RT-PCR analysis of gene expression levels

**Figure 4:**
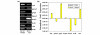
**Differences in *vim*, *krt8*, *krt19*, *hspa4*, *gstp1*, *gstm3*, and *ckb *between MCF-7-MEK5 and MCF-7-VEC cells**. **(a) **Regular RT-PCR assay. **(b) **Real-time PCR assay was used to quantitate relative mRNA expression between MCF-7-VEC and MCF-7-MEK5 cells. The normalized expression folds in MCF-7-MEK5 cells were 814.15 ± 145.23 (*vim*), 0.01 ± 0.00 (*krt8*), 0.00 ± 0.00 (*krt19*, undetected), 1.51 ± 0.56 (*hspa4*), 40,637.45 ± 15,815.03 (*gstp1*), 0.13 ± 0.01 (*gstm3*), and 38.59 ± 7.87 (*ckb*) compared with MCF-7-VEC cells. The gene *actb *was used as an internal control. MEK, mitogen-activated protein kinase kinase.

The differences in levels of VIM, KRT8, KRT19, GSTP1, GSTM3, and CKB protein expression between MCF-7-MEK5 and MCF-7-VEC cells were further confirmed at the gene expression level by RT-PCR. As shown in Figure 4a, the expression levels of *vim*, *gstp1*, and *ckb *mRNA increased markedly, whereas those of *krt8 *and *krt19 *in MCF-7-MEK5 cells were decreased compared with MCF-7-VEC cells. The results were further quantified by real-time PCR. The normalized expression fold changes in MCF-7-MEK5 cells were 814.15 ± 145.23 (*vim*), 0.01 ± 0.00 (*krt8*), 0.00 ± 0.00 (undetected; *krt19*), 1.51 ± 0.56 (*hspa4*), 40,637.45 ± 15,815.03 (*gstp1*), 0.13 ± 0.01 (*gstm3*) and 38.59 ± 7.87 (*ckb*) as compared with MCF-7-VEC cells (Figure 4b). These results indicate that the expression levels of proteins identified by proteomic analysis in MCF-7-MEK5 and MCF-7-VEC cells are consistent with corresponding gene expression levels.

### MEK5 expression upregulates mRNA levels of EMT regulating genes

**Figure 5:**
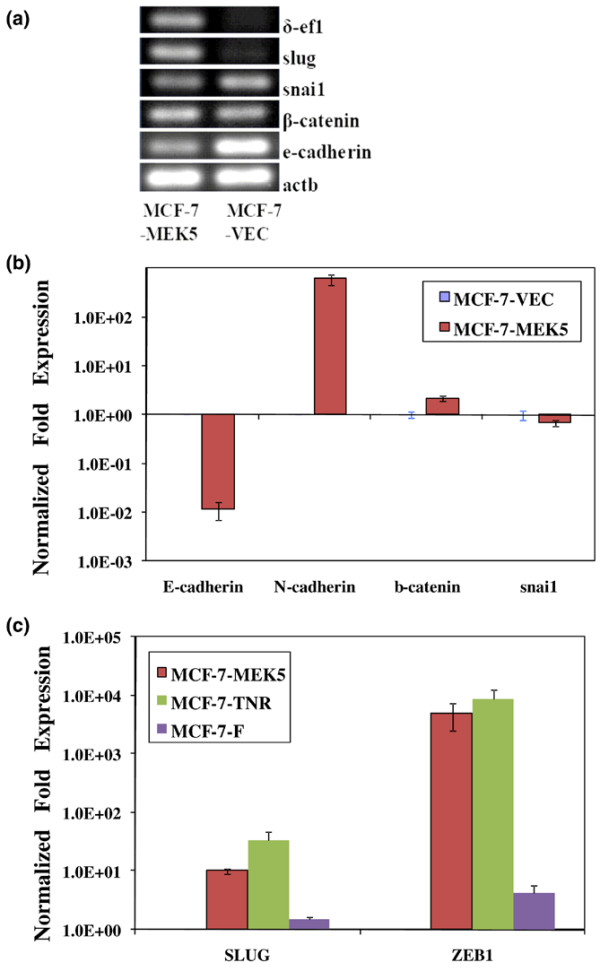
**The differences in genes involved in EMT in MCF-7-MEK5 and MCF-7-VEC cells**. **(a) **With RT-PCR assay, the genes slug, δ-ef1, and β-catenin were upregulated, whereas E-cadherin and snai1 were downregulated in MCF-7-MEK5 cells compared with MCF-7-VEC cells. **(b) **With real-time PCR, relative expression levels for N-cadherin, E-cadherin, β-catenin, and snail were analyzed in MCF-7-VEC and MCF-7-MEK5 cells. The gene *actb *was used as an internal control. **(c) **MCF-7, MCF-7-TNR, MCF-7F, and MCF-7-MEK5 cells were analyzed for expression of SNAI2 (slug) or ZEB1 (δEF-1) by real-time PCR. Expression was normalized to MCF-7 cells, with *actb *used as an internal control. MEK, mitogen-activated protein kinase kinase.

Because the observed upregulation of vimentin in the MCF-7-MEK5 cells was suggestive of EMT, we sought initially to examine the expression levels of known EMT markers in MCF-7-VEC and MCF-7-MEK5 cells (Figure 5). Analysis of E-cadherin expression by RT-PCR (Figure 5a) and quantitative PCR (Figure 5b) demonstrated a consistent downregulation in the MCF-7-MEK5 cells as compared with the MCF-7-VEC cells. N-cadherin, a known EMT marker, was upregulated in the MCF-7-MEK5 cells (599.6 ± 147.9) as compared with MCF-7-VEC cells (Figure 5b). SNAI2 (slug), SNAI1 (snail) and ZEB1 (δEF-1) are known regulators of EMT. The genes SNAI2 (slug; 10.05 ± 0.91), ZEB1 (4,866.96 ± 2,360), and β-catenin (2.16 ± 0.27) were upregulated in MCF-7-MEK5 cells compared with MCF-7-VEC cells (Figure 5b). For comparison purposes, we also analyzed expression of these EMT regulating genes in additional drug resistant MCF-7 cell systems. The TNF-resistant MCF-7-TNR cells exhibited an increase in SNAI2 (34.44 ± 12.38) and ZEB1 (8,574.28 ± 3,820) as compared with MCF-7-VEC cells. Additionally, the fulvestrant resistant MCF-7-F cells exhibited a less pronounced increase in SNAI2 (1.49 ± 0.13) and ZEB1 (4.34 ± 1.3; Figure 5c).

### MEK5 expression promotes an EMT phenotype

**Figure 6:**
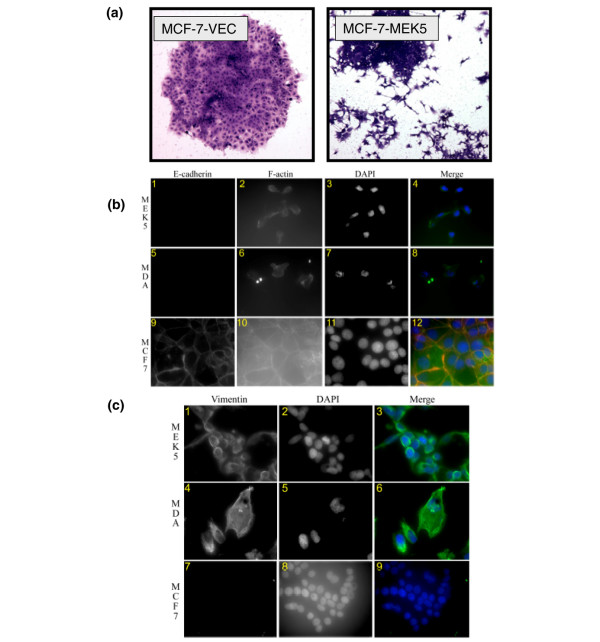
**Morphologic and immunofluorescence characterization of EMT in MCF-7-MEK5 cells**. **(a) **Morphologic comparison of MCF-7-VEC and MCF-7-MEK5 cells stained by Crystal violet assay. Representative colonies from clonogenicity assay were visualized after crystal violet staining for morphology. **(b) **MCF-7, MCF-7-MEK5, and MDA-MB231 cells were cultured in eight-well chamber slide for 48 hours. Indirect immunofluorescence was carried out to examine the expression of E-cadherin and F-actin, as described in the Materials and methods section. The nucleus was counter-stained with DAPI. Subpanels 1 through 4 are representative images of E-cadherin (1), F-actin (2), nucleus (3), and a merge of all three (4) in MCF-7-MEK5 (MEK5) cells. Subpanels 5 through 8 are representative images of E-cadherin (5), F-actin (6), nucleus (7), and a merge of all three (8) in MDA-MB231 (MDA) cells. Subpanels 9 to 12 were representative images of E-cadherin (9), F-actin (10), nucleus (11), and a merge of all three (12) in MCF-7 cells. Pseudocolors were assigned as follows: red, E-cadherin; green, vimentin; green, F-actin; and blue, nucleus. **(c) **MCF-7, MCF-7-MEK5, and MDA-MB231 cells were cultured in eight-well chamber slide for 48 hours. Indirect immunofluorescence was carried out to examine the expression of vimentin, as described in the Materials and methods section. The nucleus was counter-stained with DAPI. Subpanels 1 through 3 are representative images of vimentin (1), nucleus (2), and a merge of the two (3) in MCF-7-MEK5 (MEK5) cells. Subpanels 4 through 6 are representative images of vimentin (4), nucleus (5), and a merge of the two (6) in MDA-MB231 (MDA) cells. Panels 7 through 9 are representative images of vimentin (7), nucleus (8), and a merge of the two (9) in MCF-7 cells. Pseudocolors were assigned as follows: red, E-cadherin; green, vimentin; green, F-actin; and blue, nucleus. MEK, mitogen-activated protein kinase kinase.

Images of stained MCF-7-VEC and MCF-7-MEK5 cell colonies revealed distinct differences in cell and colony morphology (Figure 6a). MCF-7-VEC cells formed contiguous colonies with distinct colony borders. The MCF-7-VEC cells exhibited a classic epithelial cell morphology with intact cell-to-cell contact. In contrast, MCF-7-MEK5 cells exhibited mesenchymal cell morphology with a more dispersed colony appearance and incompletely defined colony borders, both suggestive of an EMT phenotype. To investigate the potential role of MEK5 signaling in EMT, MCF-7-VEC and MCF-7-MEK5 cells were stained for epithelial markers E-cadherin and F-actin (Figure 6b) or the mesenchymal marker vimentin (Figure 6c) using immunofluorescence staining. MDA-MB-231 cells, which are known to exhibit an EMT phenotype, were used as a positive control for loss of E-caherin and upregulation of vimentin. MCF-7-VEC cells exhibited typical epithelial staining pattern of E-cadherin, which is predominantly expressed on the cell membrane. Consistently, F-actin in MCF-7-VEC cells was organized in an epithelial pattern, in that the majority of F-actin existed in a cortical pattern on the cell membrane along with minor existence in focal adhesion. In contrast, epithelial signature expression and organization of E-cadherin and F-actin were absent in MCF-7-MEK5 and MDA-MB-231 cells. Conversely, MCF-7-MEK5 and MDA-MB-231 cells, but not MCF-7-VEC cells, exhibited robust staining of vimentin, which is typically observed in mesenchymal cells. These results suggest loss of epithelial markers and acquisition of mesenchymal markers in MCF-7-MEK5 and MDA-MB-231 cells.

### RNA interference knock-down of Erk5 abrogates expression of EMT regulating genes in MCF-7-MEK5 cells

**Figure 7:**
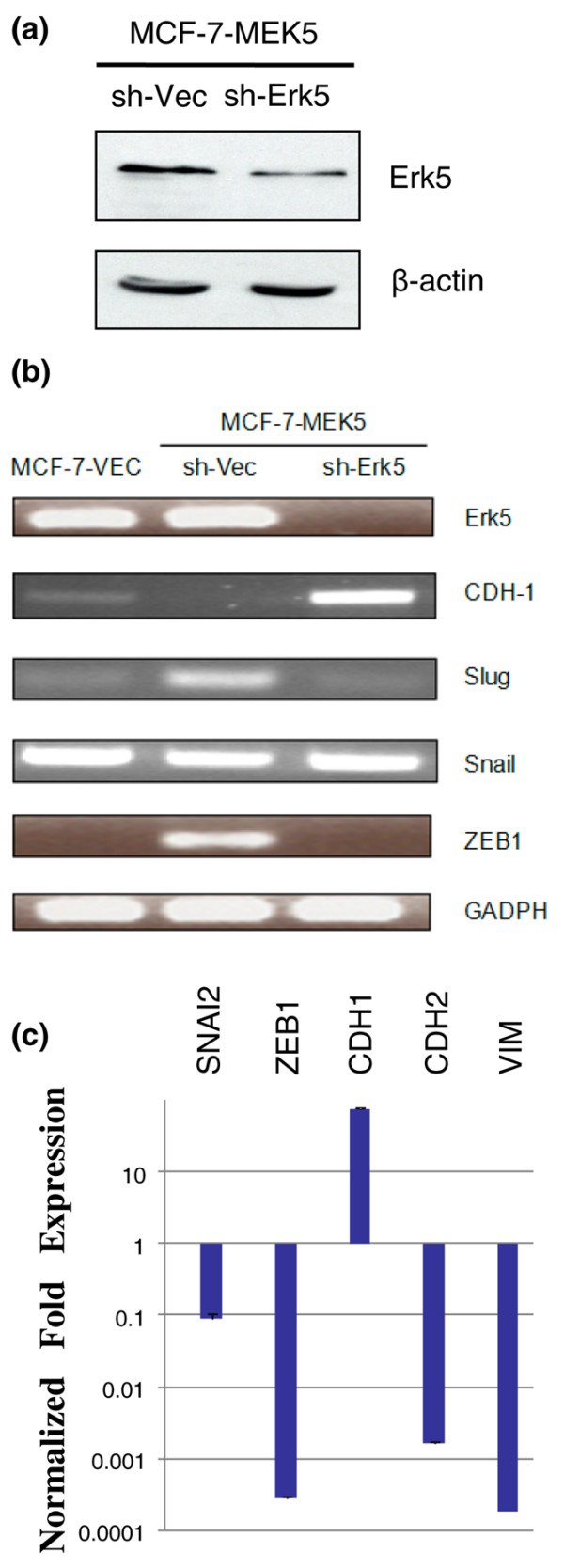
**Erk5-RNA interference partially suppresses MCF7-MEK5 EMT gene expression**. **(a) **MCF-7-MEK5 cells were transfected with pSUPER-scrambled (sh-VEC) or pSUPER-Erk5-RNAi (sh-Erk5) and harvested 24 hours later for Western blot analysis of Erk5 expression with actin as control. **(b) **MCF-7-MEK5 cells with sh-VEC or sh-Erk5 were harvested for RNA isolation and RT-PCR analysis of expression of MEK5, Erk5, E-cadherin, SNAI1, SNAI2, ZEB1, and GAPDH. MCF-7-VEC cells were analyzed for comparison purposes. **(c) **MCF-7-MEK5 cells expressing either sh-VEC or sh-Erk5 were harvested for RNA isolation and real-time PCR analysis for expression of SNAI2, ZEB1, CDH1, CDH2, and VIM. The data are presented as mean ± standard error of the mean from three independent experiments.

Erk5 is the immediate downstream target of MEK5. We used a short hairpin RNA (shRNA) interference strategy to disrupt Erk5 expression in MCF-7-MEK5 cells and to test effects on EMT gene regulation (Figure 7). MCF-7-MEK5 cells were transfected with pSUPER-scrambled or sSUPER-Erk5. We observed a decrease in both protein (Figure 7a) and mRNA levels of Erk5 (Figure 7b). shRNA-Erk5 cells exhibited a restoration of E-cadherin levels and reduced ZEB1 and SNAI2 expression. Quantitative PCR revealed that shRNA-Erk5 expression restored E-cadherin levels and reversed expression of slug, ZEB1, N-cadherin, and vimentin expression (Figure 7c).

## Discussion

Our proteomic analysis of two MCF-7 human breast cancer cell lines revealed at least seven differentially expressed protein spots in two-dimensional gels, which may contribute to the mechanisms of MEK5/Erk5-mediated TNF-α resistance. These proteins are known to play various roles in cellular processes, including detoxification, proliferation, metabolism, and cytoskeletal organization. It is well known that human cytosolic glutathione-S-transferases, which play important roles in cellular detoxification pathways, are of direct relevance to many drug-resistance tumors [46-53]. At least one of these proteins is also implicated in EMT, suggesting a possible connection between TNF-α resistance and EMT. Both VIM and KRT belong to intermediate filaments, which are considered to be the principle cytoskeletal proteins in mammalian cells [54]. It is believed that the over-expression of VIM, which is related to poor prognosis in breast cancer patients presenting with metastasis potential [55,56], results in a more invasive capacity of breast cancer cells *in vitro *and *in vivo *[21,57,58]. Kokkinos and colleagues [21] proposed that the intermediate filament transformed from a KRT-rich to a VIM-rich network in the process of EMT in cancers [21]. Previous studies have shown that MCF-7 breast cancer cells do not typically express VIM but exhibit strong expression of KRT; the acquisition of VIM expression and the loss of KRT19 expression were associated with adriamycin-resistant MCF-7 cells compared with their parental cells [11]. Our results show increased VIM expression and decreased KRT19 and KRT8 expression in TNF-α resistant MCF-7 cells.

These findings, consistent with the cytoskeletal reorganization seen in both EMT and drug resistance, provoked further evaluation of the EMT markers snail, slug, δ-ef1, E-cadherin, and N-cadherin in the TNF-α resistant MCF-7 cells. The profound morphologic changes and enhanced invasive capabilities of EMT in various cancers are thought to be regulated by several transcription factors, including SNAI1 (snail), SNAI2 (slug), and ZEB1 (δ-ef1). Increased expression of snail and slug has been reported in invasive compared with noninvasive breast tumors and associated with lymph node metastases [59]. Increased levels of δ-ef1, also seen in invasive breast tumors, have been correlated with de-differentiation [60]. These transcription factors, known to regulate EMT in development, have all been shown to repress E-cadherin, which is the primary cell adhesion molecule in epithelial tissue [61-65]. The loss of epithelial cell-to-cell adhesion through decreased expression of E-cadherin is the hallmark of EMT that permits acquisition of a motile phenotype [65]. Interestingly, N-cadherin has been demonstrated to correlate with increased invasion and migration in breast carcinomas *in vitro*, regardless of E-cadherin status [66,67]. Further *in vivo *studies have shown that N-cadherin enhances metastasis of breast tumors via Erk signaling [68]. In this study we observed a decrease in E-cadherin expression and an increase in N-cadherin expression in the MCF-7-MEK5 cells. Additionally, slug and δ-ef1, but not snail, were significantly increased in the MCF-7-MEK5 cell line.

## Conclusion

In summary, differentially expressed proteins have been identified by proteome and gene expression analyses, suggesting that upregulation of VIM/*vim*, GSTP1/*gstp1 *and CKB/*ckb*, and downregulation of KRT8/*krt8 *and KRT19/*krt19 *are related to MEK5/Erk5-mediated TNF-α resistance in an established MCF-7 cell line. Further analyses of this cell line indicated expression of an EMT phenotype, suggesting an association between MEK5/Erk5-mediated EMT and TNF-α resistance. Additional studies are needed to clarify the functions and involvement of these proteins in the mechanisms of MEK5/Erk5-mediated TNF-α resistance and EMT in human breast cancers.

## Abbreviations

CHAPS: 3-[(3-Cholamidopropyl)dimethylammonio]-1-propanesulfonate; CKB: creatine kinase B-type; 2-DE: two-dimensional electrophoresis; DTT: dithiothreitol; EMT: epithelial-mesenchymal transition; Erk: extracellular signal-regulated kinase; GSTM3: glutathione-S-transferase Mu 3; GSTP1: glutathione-S-transferase P; HSP: heat shock protein; IPG: immobilized pH gradient; KRT: keratin; MAPK: mitogen-activated protein kinase; MEK: mitogen-activated protein kinase kinase; PBS: phosphate-buffered saline; RT-PCR: reverse transcription polymerase chain reaction; shRNA: short hairpin RNA; VIM: vimentin; ZEB1: δ-EF1.

## Competing interests

The authors declare that they have no competing interests.

## Authors' contributions

CZ performed 2-DE and RT-PCR, interpreted data, and drafted the manuscript. AMN performed quantitative PCR for EMT gene expression and drafted the manuscript. WX performed RT-PCR. QZ carried out high performance liquid chromatography-tandem mass spectrometry based protein identification and database search. YT performed Western blot analysis. MB conducted RT-PCR analysis. SE generated the MEK5 stable cells and performed colony assays. YZ designed, generated, and validated pSUPER-shRNA-Erk5. LB conducted quantitative PCR analysis of gene expression. DY and CBW conducted RT-PCR and colony assays. RS, JAM, BSB, TEW, and KPN participated in experimental design and interpretation. BS performed immunofluorescence. MEB and GW co-designed the study, directed research efforts, and critically revised the manuscript.

## Supplementary Material

Additional file 1 A Word file containing a table listing the primer sequences used in all RT-PCR experiments. Click here for file

Additional file 2 A Word file containing a table listing all identified proteins that were differentially expressed in MCF-7-MEK5 versus MCF-7-VEC breast cancer cells. Click here for file
