# Supplementary material for: Proteomic analysis of tumor necrosis factor-α resistant human breast cancer cells reveals a MEK5/Erk5-mediated epithelial-mesenchymal transition phenotype
Source: Breast Cancer Res. 2008 Dec 16;10(6):R105. doi: 10.1186/bcr2210 (PMC2656902; doi:10.1186/bcr2210)
Supplement: Additional file 1 — A Word file containing a table listing the primer sequences used in all RT-PCR experiments. [file bcr2210-S1.doc]

**Additional Data File 1:**

**Primer pairs in the performance of RT-PCR**

| **Gene Name** | **Gene ID** | **Primer Sequence (5’ → 3’)** | **Product Length (bp)** |
| --- | --- | --- | --- |
| actb | NM_001101.2 | F: GCCAACACAGTGCTGTCT | 114 |
|  |  | R: AGGAGCAATGATCTTGATCTT |  |
| vim | NM_003380.2 | F: AGATGGCCCTTGACATTG AG | 132 |
|  |  | R: CCAGAGGGAGTGAATCCAGA |  |
| krt8 | NM_002273.2 | F: TCGAGAGCTACATCAACAACCTTAG | 139 |
|  |  | R: CGCTTATTGATCTCATCCTCATACT |  |
| krt19 | NM_002276.3 | F: ACTACAGCCACTACTACACGACCAT | 136 |
|  |  | R: GTCTCAAACTTGGTTCGGAAGTC |  |
| hspa4 | NM_002154.3 | F: GAAAATGAGGAGCCAATGGA | 102 |
|  |  | R: TGTCTGCTGCTGTTGCTCTT |  |
| gstp1 | NM_000852.2 | F: ACCTCCGCTGCAAATACATC | 109 |
|  |  | R: GACAGCAGGGTCTCAAAAGG |  |
| gstm3 | NM_000849.3 | F: TCCTGGAGTTCACGGATACC | 148 |
|  |  | R: TTGTTCTTCCCATCCAGGAG |  |
| ckb | NM_001823.3 | F: GGCAACATGAAGGAGGTGTT | 124 |
|  |  | R: ATGGGCAGGTGAGGATGTAG |  |
| e-cadherin | NM_004360.2 | F: GAACGCATTGCCACATACAC | 120 |
|  |  | R: GAATTCGGGCTTGTTGTCAT |  |
| β-catenin | NM_001904.2 | F: CCATTCCATTGTTTGTGCAG | 105 |
|  |  | R: CTTCTGCAGCTTCCTTGTCC |  |
| slug | NM_003068.3 | F: CGTTTTCCAGACCCTGGTT | 122 |
|  |  | R: CTGCAGATGAGCCCTCAGA |  |
| δ-ef1 | NM_030751.2 | F: GTCCAAGAACCACCCTTGAA | 125 |
|  |  | R: TTTTTGGGCGGTGTAGAATC |  |
| snai1 | NM_005985.2 | F: GCGAGCTGCAGGACTCTAAT | 135 |
|  |  | R: GGACAGAGTCCCAGATGAGC |  |
| n-cadherin | NM_001792 | F: CTCCTATGAGTGGAACAGGAACG |  |
|  |  | R: TTGGATCAATGTCATAATCAAGTGCTGTA |  |

F=forward; R=reverse.
