# Supplementary material for: Proteomic analysis of tumor necrosis factor-α resistant human breast cancer cells reveals a MEK5/Erk5-mediated epithelial-mesenchymal transition phenotype
Source: Breast Cancer Res. 2008 Dec 16;10(6):R105. doi: 10.1186/bcr2210 (PMC2656902; doi:10.1186/bcr2210)
Supplement: Additional file 2 — A Word file containing a table listing all identified proteins that were differentially expressed in MCF-7-MEK5 versus MCF-7-VEC breast cancer cells. [file bcr2210-S2.doc]

**Additional Data File 2:**

**List of all identified proteins that were differentially expressed in MCF-7MEK5 vs MCF-7VEC breast cancer cells**

| **Differential Expressed Proteins** | **MW (Theory)** | **MW (exp)** | **PI (Theory)** | **PI (Exp)** | **MCF-7 MEK5** | **MCF-7 VEC** | **Expression Ratio** |
| --- | --- | --- | --- | --- | --- | --- | --- |
|  | (D) | (KD) |  |  |  |  | **Mek5/Vec** |
| Gene_Symbol=KRT13 Isoform 1 of Keratin, type I cytoskeletal 13 | 49556 | 50 | 4.76 | 5.6 | 1981.5 | 421135.3 | 0 |
| **Gene_Symbol=KRT19 Keratin, type I cytoskeletal 19** | **44066** | **46** | **4.9** | **5.2** | **23482.9** | **1699859** | **0.01** |
| Gene_Symbol=TUBB2C Tubulin beta-2C chain | 49800 | 52 | 4.65 | 5.3 | 902683.9 | 512719.6 | 1.76 |
| Gene_Symbol=WWC2 WW, C2 and coiled-coil domain-containing protein 2 | 109914 | 80 | 5.13 | 5.3 | Not detected | 40987.4 | 0 |
| Gene_Symbol=EIF5A2 Eukaryotic translation initiation factor 5A-2 | 16783 | 17 | 5.28 | 5.6 | 48460.2 | 180560.3 | 0.27 |
| **Gene_Symbol=GSTM3 Glutathione S-transferase Mu 3** | **26543** | **26** | **5.25** | **5.7** | **42921.6** | **114505.5** | **0.37** |
| Gene_Symbol=UCHL1 Ubiquitin carboxyl-terminal hydrolase isozyme L1 | 24809 | 25 | 5.22 | 5.6 | 10063.5 | 26325.9 | 0.38 |
| Gene_Symbol=DKFZp686D0972 hypothetical protein LOC345651 | 41977 | 41 | 5.29 | 5.6 | 48685.6 | 218549.8 | 0.22 |
| Gene_Symbol=KRT71 Keratin-71 | 57257 | 40 | 6.25 | 5.8 | 8350.7 | 54048.6 | 0.15 |
| Gene_Symbol=KRT18 49 kDa protein | 48793.9 |  | 5.45 |  | 20374 | 258442.8 | 0.08 |
| Gene_Symbol=KRT18 49 kDa protein | 48793 | 49 | 5.45 | 5.7 | 296675 | 2281260 | 0.13 |
| **Gene_Symbol=VIM Vimentin** | **53620** | **55** | **4.91** | **5.2** | **1985265** | **928259.7** | **2.14** |
| Gene_Symbol=LMNB1 Lamin-B1 | 66368.6 | 74 | 4.96 | 5.4 | 214469.6 | 112091.2 | 1.91 |
| Gene_Symbol=HNRPK Isoform 1 of Heterogeneous nuclear ribonucleoprotein K | 50945 | 50 | 5.26 | 5.6 | Not detected | 69129 | 0 |
| **Gene_Symbol=HSPA4 Heat shock 70 kDa protein 4** | **94241** | **100** | **5.03** | **5.3** | **37598.4** | **20813.1** | **1.81** |
| Gene_Symbol=PRDX2 Peroxiredoxin-2 | 21879 | 22 | 5.59 | 5.8 | 11282.8 | 322996.1 | 0.03 |
| **Gene_Symbol=GSTP1 Glutathione S-transferase P** | **23342** | **23** | **5.32** | **5.8** | **190084.8** | **35156.7** | **5.41** |
| Gene_Symbol=PSME2 Proteasome activator complex subunit 2 | 27345 | 29 | 5.33 | 5.8 | 18166.4 | 102908.3 | 0.18 |
| Gene_Symbol=NDUFS3 NADH dehydrogenase [ubiquinone] iron-sulfur protein 3, mitochondrial precursor | 30223 | 29 | 7.35 | 6.8 | 15683.3 | 560291.5 | 0.03 |
| Gene_Symbol=PPA1 Inorganic pyrophosphatase | 32640 | 34 | 5.47 | 5.8 | 20510.7 | 76640.5 | 0.27 |
| Gene_Symbol=PPA1 Inorganic pyrophosphatase | 32640 | 35 | 5.47 | 5.75 | 102343.9 | 32499.7 | 3.15 |
| Gene_Symbol=RPLP0 60S acidic ribosomal protein P0 | 34252 | 35 | 5.63 | 5.8 | 251771.7 | 78396.8 | 3.21 |
| **Gene_Symbol=CKB Creatine kinase B-type** | **42618** | **45** | **5.65** | **5.7** | **1112306** | **19286** | **57.67** |
| Gene_Symbol=KRT18 49 kDa protein | 48793 | 49 | 5.45 | 5.75 | 23248.3 | 108396.6 | 0.21 |
| **Gene_Symbol=KRT8 Keratin, type II cytoskeletal 8** | **53672** | **50** | **5.38** | **5.8** | Not detected | **384924.7** | **0** |
| Gene_Symbol=KRT18 49 kDa protein | 48793 | 49 | 5.45 | 5.8 | Not detected | 123604.4 | 0 |
| Gene_Symbol=GFAP Isoform 1 of Glial fibrillary acidic protein, astrocyte | 49850 | 50 | 5.3 | 5.8 | 11091.7 | 1562987 | 0.01 |
| Gene_Symbol=CCT5 T-complex protein 1 subunit epsilon | 59633.8 | 58 | 5.34 | 5.6 | 100714.6 | 205018 | 0.49 |
| Gene_Symbol=HSPA1L Heat shock 70 kDa protein 1L | 70332 | 74 | 5.67 | 5.7 | 272106.7 | 60113.3 | 4.53 |
| Gene_Symbol=HSPA1B;HSPA1A Heat shock 70 kDa protein 1 | 69999 | 70 | 5.35 | 5.75 | 3456580 | 1143.2 | 3023.68 |
| Gene_Symbol=PLS3 plastin 3 | 70767 | 73 | 5.3 | 5.8 | 245806.5 | 65013.8 | 3.78 |
| Gene_Symbol=SOD1 16 kDa protein | 16113 | 17 | 5.86 | 5.88 | 255317.2 | 127505.9 | 2.00 |
| Gene_Symbol=NME1 Nucleoside diphosphate kinase A | 17138 | 18 | 5.78 | 6 | 121617.6 | 51433.2 | 2.36 |
| Gene_Symbol=PSME1 Proteasome activator complex subunit 1 | 28706 | 28 | 5.7 | 5.9 | 30736.2 | 207150.1 | 0.15 |
| Gene_Symbol=LDHB L-lactate dehydrogenase B chain | 36616 | 35 | 5.66 | 5.9 | 709866.3 | 7025.5 | 101.04 |
| Gene_Symbol=ALDH2 Aldehyde dehydrogenase, mitochondrial precursor | 56346.6 | 50 | 6.67 | 5.9 | 82991 | 46841.8 | 1.77 |
| Gene_Symbol=ALB ALB protein | 71658 | 70 | 6.4 | 6 | 28451.6 | 62255.9 | 0.46 |
| Gene_Symbol=CDH4 Cadherin-4 precursor | 100218 | 100 | 4.5 | 5.9 | 51429.4 | 15189.2 | 3.39 |
| Gene_Symbol=S100A11 Protein S100-A11 | 11733 | 12 | 7.01 | 6.4 | 18755.6 | 298904.2 | 0.06 |
| Gene_Symbol=HSPB1 Heat-shock protein beta-1 | 22769 | 27 | 5.97 | 6.2 |  | 503204.5 | 0 |
| Gene_Symbol=RAB14 Ras-related protein Rab-14 | 23882 | 24 | 5.81 | 6.3 | 29170.2 | 74418.7 | 0.39 |
| Gene_Symbol=SLC25A24 Solute carrier family 25 member 24 | 53321 | 48 | 5.95 | 6.3 |  | 49319.4 | 0 |
| Gene_Symbol=PRDX6 Peroxiredoxin-6 | 25020 | 25 | 5.97 | 6.6 | 122176.6 | 227121.8 | 0.54 |
| Gene_Symbol=PRPS1 Ribose-phosphate pyrophosphokinase I | 34812 | 29 | 6.54 | 6.55 | 12548.2 | 19655.7 | 0.64 |
| REFSEQ:XP_001133946;XP_001133947;XP_001133949;X | 26926 | 26 | 8.08 | 6.5 | 20902.9 | 104143.1 | 0.2 |
| Gene_Symbol=VIL2 Ezrin | 69645 | 74 | 5.89 | 6.4 | 142259.3 | 49313.6 | 2.88 |
| VEGA:OTTHUMP00000167 | 38198 | 39 | 6.35 | 6.6 | 74011.3 | 45503.2 | 1.63 |
| Gene_Symbol=CCT6A T-complex protein 1 subunit zeta | 57988 | 60 | 6.23 | 6.6 | 133733.8 | 75822.4 | 1.76 |
| Gene_Symbol=PHGDH D-3-phosphoglycerate dehydrogenase | 56615 | 53 | 6.29 | 6.7 | 139243.9 | 67184.2 | 2.07 |
| Gene_Symbol=G6PD Isoform Long of Glucose-6-phosphate 1-dehydrogenase | 63787 | 55 | 6.44 | 6.7 | 52538.3 | 141647.8 | 0.37 |
| Gene_Symbol=XRCC6 70 kDa protein | 69827 | 70 | 6.21 | 6.6 | 82335.5 | 48321.1 | 1.70 |
| Gene_Symbol=ALDOC Fructose-bisphosphate aldolase C | 39432 | 39 | 6.44 | 6.9 | 82201 | 130505.8 | 0.63 |
| Gene_Symbol=KRT2 Keratin, type II cytoskeletal 2 epidermal | 65826 | 55 | 8.03 | 6.9 | 8252 | 116011.6 | 0.07 |
| Gene_Symbol=EEF2 Elongation factor 2 | 95278 | 90 | 6.4 | 6.9 | 67170.1 | 18392.1 | 3.65 |
| Gene_Symbol=RAN GTP-binding nuclear protein Ran | 24408 | 25 | 7.3 | 7.22 | 94323.5 | 39458.4 | 2.39 |
| Gene_Symbol=ENO1 Isoform alpha-enolase of Alpha-enolase | 47140 | 45 | 7.16 | 7.15 | 282521.4 | 6105.8 | 46.27 |
